# HAMLET effect on cell death and mitochondrial respiration in colorectal cancer cell lines with KRAS/BRAF mutations

**DOI:** 10.1007/s00432-023-04777-0

**Published:** 2023-04-26

**Authors:** Justas Žilinskas, Darius Stukas, Aldona Jasukaitienė, Jurgita Šapauskienė, Rasa Banienė, Sonata Trumbeckaitė, Saulius Švagždys, Marco Cicciu, Žilvinas Dambrauskas, Antanas Gulbinas, Algimantas Tamelis

**Affiliations:** 1 Department of Surgery, Medical Academy, Faculty of Medicine, Lithuanian University of Health Sciences, Eivenių Street 2, 50161 Kaunas, Lithuania; 2 Institute of Digestive Research, Medical Academy, Faculty of Medicine, Lithuanian University of Health Sciences, Kaunas, Lithuania; 3 Department of Biochemistry, Lithuanian University of Health Sciences, Kaunas, Lithuania; 4 Laboratory of Biochemistry, Neuroscience Institute, Lithuanian University of Health Sciences, Kaunas, Lithuania; 5 Department of Biomedical and Dental Sciences, Morphological and Functional Images, School of Dentistry, University of Messina, Messina, Italy; 6 Department of Pharmacognosy, Medical Academy, Lithuanian University of Health Sciences, Kaunas, Lithuania

**Keywords:** Colorectal cancer, Bioactive milk components, KRAS and BRAF mutation, EGFR, Mitochondrial respiration

## Abstract

**Purpose:**

Treatment of advanced colorectal cancer (CRC) depends on the correct selection of personalized strategies. HAMLET (Human Alpha-lactalbumin Made LEthal to Tumor cells) is a natural proteolipid milk compound that might serve as a novel cancer prevention and therapy candidate. Our purpose was to investigate HAMLET effect on viability, death pathway and mitochondrial bioenergetics of CRC cells with different KRAS/BRAF mutational status in vitro.

**Methods:**

We treated three cell lines (Caco-2, LoVo, WiDr) with HAMLET to evaluate cell metabolic activity and viability, flow cytometry of apoptotic and necrotic cells, pro- and anti-apoptotic genes, and protein expressions. Mitochondrial respiration (oxygen consumption) rate was recorded by high-resolution respirometry system Oxygraph-2 k.

**Results:**

The HAMLET complex was cytotoxic to all investigated CRC cell lines and this effect is irreversible. Flow cytometry revealed that HAMLET induces necrotic cell death with a slight increase in an apoptotic cell population. WiDr cell metabolism, clonogenicity, necrosis/apoptosis level, and mitochondrial respiration were affected significantly less than other cells.

**Conclusion:**

HAMLET exhibits irreversible cytotoxicity on human CRC cells in a dose-dependent manner, leading to necrotic cell death and inhibiting the extrinsic apoptosis pathway. BRAF-mutant cell line is more resistant than other type lines. HAMLET decreased mitochondrial respiration and ATP synthesis in CaCo-2 and LoVo cell lines but did not affect WiDr cells’ respiration. Pretreatment of cancer cells with HAMLET has no impact on mitochondrial outer and inner membrane permeability.

## Introduction

Approximately, 2 million new colorectal cancer (CRC) cases and 935,000 deaths were estimated to occur in 2020, accounting for about 10% of all diagnosed malignancies and cancer-related deaths worldwide. Therefore, significant bowel cancer rates are third in incidence and second in mortality (Sung et al. 2021). However, a quarter of these patients have advanced disease at the time of diagnosis, while 20% of patients will be diagnosed with metastatic at a later time (Aasebø et al. 2020).

Personalized medicine development of new active agents as an adjunct to chemotherapy has enhanced metastatic CRC (mCRC) outcomes in the 21st century. For instance, monoclonal antibodies such as bevacizumab target vascular endothelial growth factor, while cetuximab acts directly against epidermal growth factor receptor (EGFR) (Douillard et al. 2013; Cohen et al. 2021; Tougeron et al. 2013). Second, the scientific progress of patient molecular profile and heterogeneous tumor microenvironment demands complex oncological treatment decisions. For example, activating mutations in RAS, BRAF V600E induce resistance to EGFR inhibitors (Molinari et al. 2018; Oikonomou et al. 2014). Other essential agents include human EGFR 2, programmed death receptor 1 and tropomyosin receptor kinase inhibitors (Fujii et al. 2020; André et al. 2020; Cooper et al. 2020).

Regardless of therapeutic advancement, the median overall survival of selected mCRC patients improved to the extent of 20–30 months in clinical trials. In comparison, the prognosis for an unselected population from the Scandinavian cancer registry remains significantly shorter, with a median overall survival of 10–15 months. In addition, subjects from clinical trials usually have better performance status, younger age, and less comorbidity, making them incomparable to the general mCRC population (Aasebø et al. 2020; Hamers et al. 2019). Opposite survival findings from trials and registries challenge the need to develop novel therapeutic agents.

Traditionally, natural products have been the prim3

02}>M+ry origin of bioactive syntheses used in the pharmaceutical industry and traditional healthcare systems (Genovese et al. 2020). A novel promising candidate is human alpha-lactalbumin made lethal to tumor cells (HAMLET), a new type of cancer-killing molecule developed by the Lund University research group. It is a complex of two of the most abundant units in human milk: Protein alpha-lactalbumin and lipid oleic acid. Together they form a compound with a broad tumoricidal effect against cancer cells without harming mature, healthy cells (Ho et al. 2017, 2016; Arcila et al. 2011).

HAMLET independently hits multiple cell targets, including the EGFR signaling pathway. Two of the key signaling molecules of the pathway are RAS and RAF, encoded by the *KRAS* and *BRAF* genes, respectively (Kim and Bodmer 2022). Typically, these mutations occur in CRC, and their presence links to EGFR inhibitor resistance (Ho et al. 2017) revealing that HAMLET inhibits oncogenic Ras and Braf activity (Ho et al. 2016). In addition, HAMLET is known to activate mitochondria-dependent apoptosis and might interfere with mitochondrial function (Boekema et al. 2015). However, the cell death mechanism is undefined. Thus, we hypothesized that HAMLET anticancer effectiveness might be affected by different *KRAS*/*BRAF* mutational status and mitochondrial activity of CRC cell lines.

## Materials and methods

### Cell cultures and reagents

Human CRC cell lines WiDr (colorectal adenocarcinoma) and LoVo (colorectal adenocarcinoma from metastatic site) were obtained from CLS cell lines service, Germany. Caco-2 (colorectal adenocarcinoma) cell line was obtained from American Type Culture Collection (ATCC, United States). The mutation status of CRC cell lines is summarized in Table 1 (Ahmed et al. 2013). Caco-2 and LoVo cell lines were cultured in Ham’s F-12K (Kaighn’s) medium (GIBCO) supplemented with 10% fetal bovine serum (GIBCO) and 1% penicillin-streptomycin (GIBCO). WiDr cell line was cultured in 1:1 Ham’s F-12K (Kaighn’s) medium (GIBCO) and Dulbecco’s modified eagle medium (GIBCO) supplemented with 5% fetal bovine serum (GIBCO) and 1% penicillin-streptomycin (GIBCO). The cell lines were incubated at 37 °C in a 5% CO_2_ atmosphere.

**Table 1:** Colon cancer cell lines are classified by the mutation status of cancer genes

*Colorectal Cell line*	KRAS mutation	BRAF mutation
Caco-2	Wild type	Wild type
LoVo	G13D; A14V	Wild type
WiDr	wild type	V600E

Adapted from Ahmed et al. (2013)

Human alpha-lactalbumin (Cat. No. L7269) and oleic acid (Cat. No. O1383) were purchased from Sigma-Aldrich, Germany. 3-(4,5-Dimethylthiazol-2yl)-2,5-diphenyltetrazolium bromide (MTT) (Cat. No. M6494) was purchased from Thermo Fisher Scientific. Dimethyl sulfoxide (Cat. No. A944.2) was purchased from Carl Roth, Germany. The staining dyes, Flow Cellect Mito Damage Kit (Cat. No. FCCH100106) and Annexin V-PE Apoptosis detection kit (Cat. No. CBA606), were purchased from EMD Millipore, United States.

### Formation of the HAMLET complex

The HAMLET complex was formed from human alpha-lactalbumin and oleic acid using the heat-treatment method as described (Kamijima et al. 2008). Human alpha-lactalbumin was dissolved in phosphate-buffered saline and incubated at 50 °C for 15 min, shaking. After 15 min of incubation and shaking, oleic acid was added. The solution was repeatedly incubated at 50 °C for 10 min, shaking. The solution was then cooled to room temperature, and excess oleic acid was removed via centrifugation. After production, the HAMLET complex was stored at − 80 °C.

### Cell viability assay

Inhibition of cell growth in response to HAMLET was measured by MTT colorimetric assay. During the assay, HAMLET cytotoxicity was measured for 48 h by seeding cells into a 96 well plate at a density of 8 × 103 to 2 × 104 cells/well (exact concentration was cell line-dependent). The HAMLET complex was added to the cell culture 24 h after plating, and cells were further incubated for 6 h. Subsequently, the growth medium was changed, and cells were incubated further for 18 h, followed by the addition of MTT reagent. The chemical reaction with MTT took place for 3–4 h at 37 °C, and the growth medium was then removed by aspiration. Formed formazan crystals were dissolved in 100 μL dimethyl sulfoxide, and the absorption was measured at 570/620 nm. Colorimetric absorption values were compared to the control group.

### Clonogenic assay

Clonogenic assays were performed by seeding 1 × 102 to 2 × 102 cells/well in 24-well plates. After 24 h of plating, the HAMLET complex was added, and cells were incubated for 6 h in the presence or absence of different HAMLET complex concentrations. After 6 h, the culture medium was changed into a fresh culture medium without HAMLET, and the cells were incubated for 8 days. Cells were then fixed with ethanol and stained with crystal violet. The number of colonies (> 50 cells) was counted using an inverted microscope. All values were compared to the control group.

### Flow cytometric analysis

Flow cytometric analysis was performed using Guava Personal Cell Analysis Flow Cytometer (Merck, Millipore, Burlington, MA, United States) and CytoSoft 2.1.4 software. The assay was performed by seeding 1 × 105 to 1.3 × 105 cells/well. After 24 h of plating, the HAMLET complex was added, and cells were incubated for 6 h in the absence of different HAMLET complex concentrations. After 6 h, cells were detached using trypsin-EDTA without discarding the floating cells. The culture medium was removed by centrifugation, and cells were suspended in a binding buffer. The cells were stained with annexin V-PE and 7-AAD dyes and measured by flow cytometry.

### RNA extraction and real-time polymerase chain reaction (RT-PCR)

Total RNA extraction was performed from cultured cells using the RNA extraction kit (Abbexa) according to the manufacturer’s protocol. Purified RNA was quantified and assessed for purity by UV spectrophotometry (NanoDrop). cDNA was generated from 2 μg of RNA with High-Capacity cDNA Reverse Transcription Kit, (Applied Biosystems). The amplification of specific RNA was performed in a 20 μl reaction mixture containing 2 μl of cDNA template, 1X PCR master mix, and the primers. The PCR primers used for the detection of BIRC2 (Hs01112284_m1), BIRC3 (Hs00985031_g1), BIRC5 (Hs00153353_m1), XIAP (Hs00745222_s1), APAF-1 (Hs00559441_m1) and housekeeping gene GAPDH (Hs02758991_g1) were from Applied Biosystems.

### Western blot analysis

Lysates from cells were prepared using radioimmunoprecipitation lysis buffer (Abcam, Cambridge, UK) containing protease inhibitors (Roche Diagnostics, Basel, Switzerland). A bicinchoninic acid protein assay kit (Thermo Fisher Scientific, Inc.) was used to determine the protein concentration according to the manufacturer’s protocols. Following heating at 97 °C for 5 min, protein samples (50 µg) were subjected to 4–12% SDS-PAGE and transferred to polyvinylidene fluoride membranes at 30 V for 50 min. Membranes were blocked with a blocking buffer (20% diluent A, 30% diluent B; Western Breeze Blocker/Diluent; Invitrogen; Thermo Fisher Scientific, Inc.) at room temperature for 30 min and incubated with the primary antibodies rabbit anti-cas9 (dilution 1:1000; cat. no., PA5-19904; Invitrogen; Thermo Fisher Scientific, Inc) and mouse anti-GAPDH (dilution, 1:3000; cat. no., AM4300; Ambion; Thermo Fisher Scientific, Inc.) at 4 °C overnight. The following day, the blots were incubated with ready-to-use secondary antibodies against rabbit (cat. no. WP20007; Invitrogen, Thermo Fisher Scientific, Inc.) or mouse immunoglobulin G (cat. no. WP20006; Invitrogen; Thermo Fisher Scientific, Inc.) for 30 min at room temperature. Chemiluminescence substrate (CDP-Star; Invitrogen; Thermo Fisher Scientific, Inc.) was added, and the ChemiDoc imaging system (Bio-Rad Laboratories, Inc.) was used for visualization. ImageJ software (version 1.48; National Institutes of Health, Bethesda, MD, USA) was used for quantification of western blots.

### Measurement of mitochondrial function in cancer cells

**Fig. 1:**
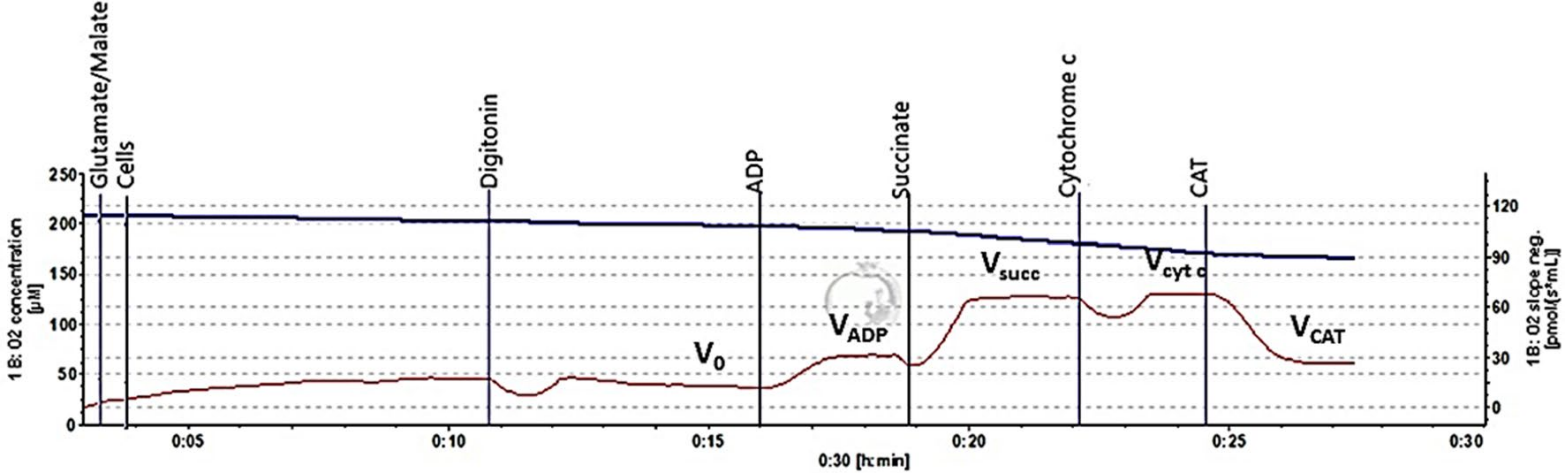
Typical trace of colon cancer cells mitochondrial respiration. Mitochondrial non-phosphorylating state 2 (V_0_) respiration rate was recorded in the medium supplemented with colorectal cancer cells (1 mln cells/2 ml) and mitochondrial Complex I substrate (5 mM glutamate + 2 mM malate). Digitonin (16 µg/ml) was added in order to permeabilize cell membrane. The state 3 respiration rate (V_ADP_) was determined following the addition of 1 mM ADP. Complex II substrate succinate (12 mM) was used to achieve maximal mitochondrial respiration (Vsucc). The effect of cytochrome c on respiration rate (indicating mitochondrial outer membrane permeability) was determined by adding 32 μM cytochrome c. Carboxyatractyloside (1 μM), an inhibitor of ADP/ATP translocator (V_CAT_), was added to evaluate the permeability of mitochondrial inner membrane

Mitochondrial respiration (oxygen consumption) rate was recorded by high-resolution respirometry system Oxygraph-2k (OROBOROS Instruments, Innsbruck, Austria) at 37 °C in the medium containing 0.5 mM EGTA, 3 mM MgCl2, 60 mM K-lactobionate, 20 mM Taurine, 10 mM KH2PO4, 20 mM HEPES, 110 mM sucrose (pH 7.1 at 37 °C). WE investigated mitochondrial functions according to a multiple substrate–inhibitor titration (Fig. 1). Digitonin (16 µg/ml) was added in order to permeabilize the cell membrane. Mitochondrial non-phosphorylating state State 2 (V_0_) respiration rate was recorded in the medium supplemented with cells and mitochondrial Complex I substrate (5 mM glutamate +2 mM malate). The state 3 respiration rate (V_ADP_) was determined after adding 1 mM ADP. Complex II substrate succinate (12 mM) was used to achieve maximal mitochondrial respiration (V_succ_). The effect of cytochrome c on respiration rate (indicating mitochondrial outer membrane permeability) was determined by adding 32 μM cytochrome c. The respiratory control index (RCI) for glutamate/malate was calculated as the ratio between V_ADP_/V_0_ respiration rate. Datlab 5 software (Oroboros Instruments) was used for real-time data acquisition and data analysis. Oxygen consumption was related to cell number (pmol/s/1 mln cells).

### Statistical analysis

GraphPad Prism 6 and SigmaPlot software were used for statistical analysis. The Mann–Whitney test was used for non-parametric data. Association between qualitative values in comparative groups was assessed by the *χ*2 test and interval and categorical by the Student’s *t* test. The level of significance was set at 0.05.

## Results

### Cell viability suppression caused by the HAMLET complex

**Fig. 2:**
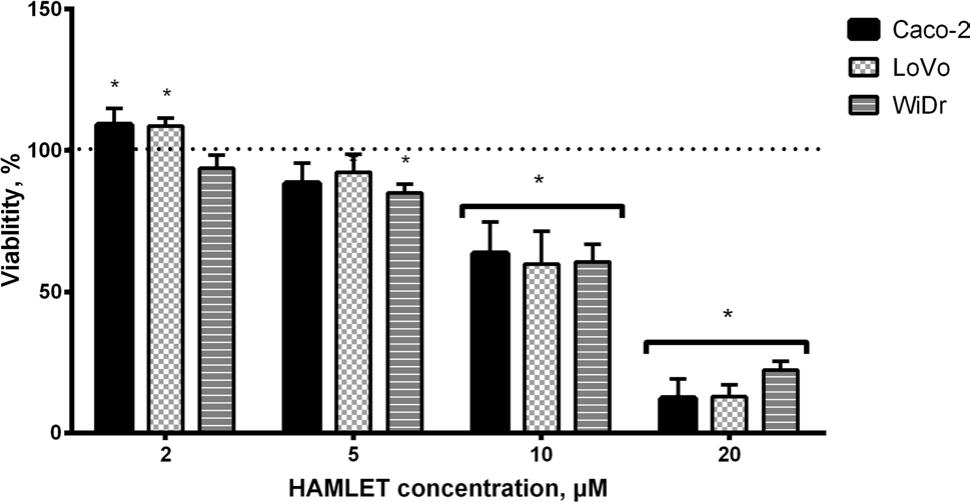
The MTT assay performed 24 h after a 6 h incubation revealed a dose-dependent response and higher WiDr cell line resistance to the 20 μM human alpha-lactalbumin made lethal to tumor cells complex. *p < 0.05: Compared to control group data (100%, dotted line). HAMLET: Human alpha-lactalbumin made lethal to tumor cells

Initially, we evaluated the effect of the HAMLET complex on the viability of CRC cell lines with different mutational statuses. We examined cell metabolism response to 2 μM, 5 μM, 10 μM and 20 μM concentrations of the HAMLET complex. All concentrations of the HAMLET complex affected all cell lines in a dose–response relationship (Fig. 2). The lowest tested concentration of HAMLET (2 μM) had a minimal effect on cell viability. Caco-2 [KRAS/BRAF wild-type (wt)] and LoVo [KRAS mutant (mt), BRAF wt] viability increased significantly to 109% and WiDr (KRAS wt, BRAF mt) had no significant change in viability. When the HAMLET complex concentration was increased to 5 μM, the viability of all cell lines decreased. However, the suppression of viability was significant only in WiDr (85%) cell line. Cells treated with 10 μM of HAMLET complex significantly decreased cell viability. When comparing the effect of 10 μM HAMLET to control (100%), Caco-2 viability was reduced to 64%, LoVo to 60%, WiDr to 61%. At the highest concentration (20 μM) of the HAMLET complex a difference between BRAF mutant and KRAS mt or WT cells was observed—WiDr cell line was more resistant to the effects of 20 μM HAMLET complex (12-12 % viability for Caco-2 and LoVo compared to 22 % viability for WiDr cell line).

### HAMLET complex effect on prolonged cell survival by clonogenic assay

**Fig. 3:**
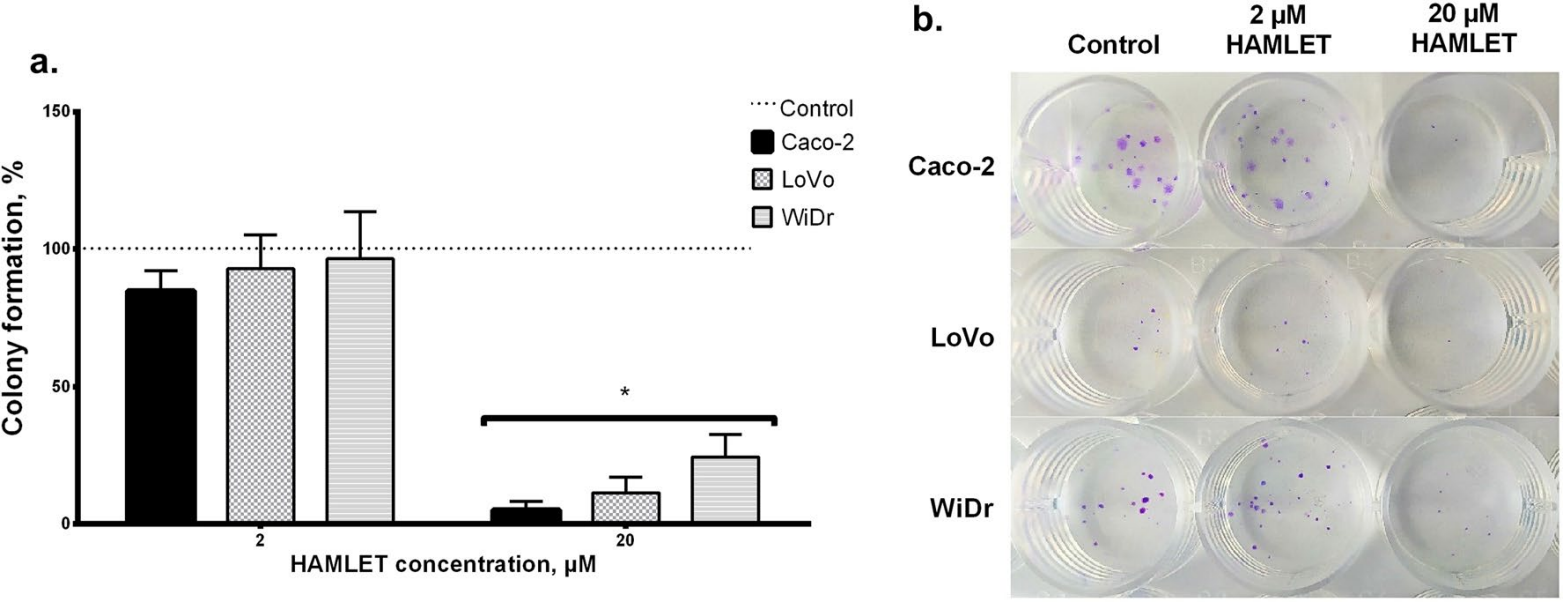
The effect of HAMLET on colony formation in different colorectal cancer cell lines. **A** Clonogenic assay performed 8 days after incubation with the HAMLET complex revealed that WiDr cell line was the most resistant to 20 μM HAMLET; **B** Representative pictures of the colony formation assay. * < 0.05: compared to control group data (100%, dotted line)

In addition to suppressing cell viability, the HAMLET complex also significantly affected colony formation (Fig. 3). The pattern of results obtained by the clonogenic assay was comparable to those obtained by MTT (Fig. 2). Similarly, BRAF mutation seemed to impact the HAMLET complex response since the WiDr cell line was the most resistant to the 20 μM HAMLET complex.

### HAMLET-induced apoptosis/necrosis signal analysis by flow cytometry

**Fig. 4:**
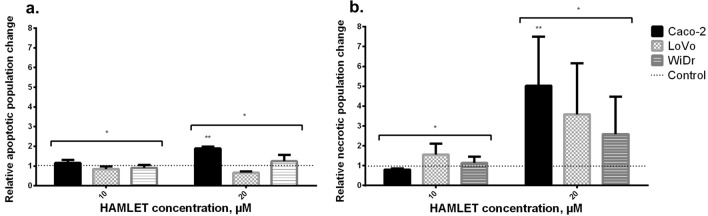
Flow cytometric analysis of HAMLET effect to different cell lines. **A** A low increase in apoptotic cell population and slight differences between cell lines; **B** a high increase in necrotic cell population in all cell lines. *p ≤ 0.05 when comparing apoptosis and necrosis of the same sample. **p < 0.05 when comparing between 10 μM and 20 μM. Control group data = 1—dotted line

The results of the MTT assay and colony formation test were confirmed by flow cytometry (Fig. 4), which showed similar tendencies of rejecting our hypothesis with HAMLET and mutational status relationship. For clarity, we present the flow cytometry outcomes of only three cell lines corresponding to a different type of mutation: Caco-2 [KRAS/BRAF wild-type (wt)], LoVo [KRAS mutant (mt), BRAF wt] and WiDr [KRAS wt, BRAF mt].

The numbers of cells undergoing necrosis or apoptosis after treatment with the 10 and 20 μM HAMLET complex were evaluated by flow cytometry. The assay showed a meager increase or even a decrease in apoptotic cell population when comparing untreated samples with samples treated with 10 or 20 μM HAMLET complex (Fig. 3A). After treatment with 10 μM HAMLET complex Caco-2 cell population increased 1.15 times, LoVo decreased 0.84 times and WiDr—0.91 when compared to control samples. Treating with 20 μM, Caco-2 apoptotic cell population increased 1.9 times, LoVo decreased 0.66 times and WiDr increased 1.25 times when compared to control. However, after the exposure of 20 μM, the increase in the necrotic cell population (Fig. 3B) was much more prominent than the apoptotic population. After treatment with 10 μM HAMLET complex Caco-2 necrotic cell population decreased 0.8 times, LoVo increased 1.55 times and WiDr—1.13 times when compared to control samples. Treating with 20 μM, Caco-2 apoptotic cell population increased 5 times, LoVo—3.59 times and WiDr—2.69 times when compared to control. Yet again, WiDr cell line was the most resistant and had the lowest increase in a necrotic cell population. The results indicate that the HAMLET complex mainly causes necrotic death in colorectal cancer cell lines.

To summarize the results in the figures (Figs. 2, 3, 4), there was no correlation between the HAMLET-induced cell death level and KRAS/BRAF mutations. However, the WiDr cell line differs from the other lines as being more resistant in terms of cell metabolism, increased necrotic cell population and colony formation.

### RT-PCR and WB analysis

**Fig. 5:**
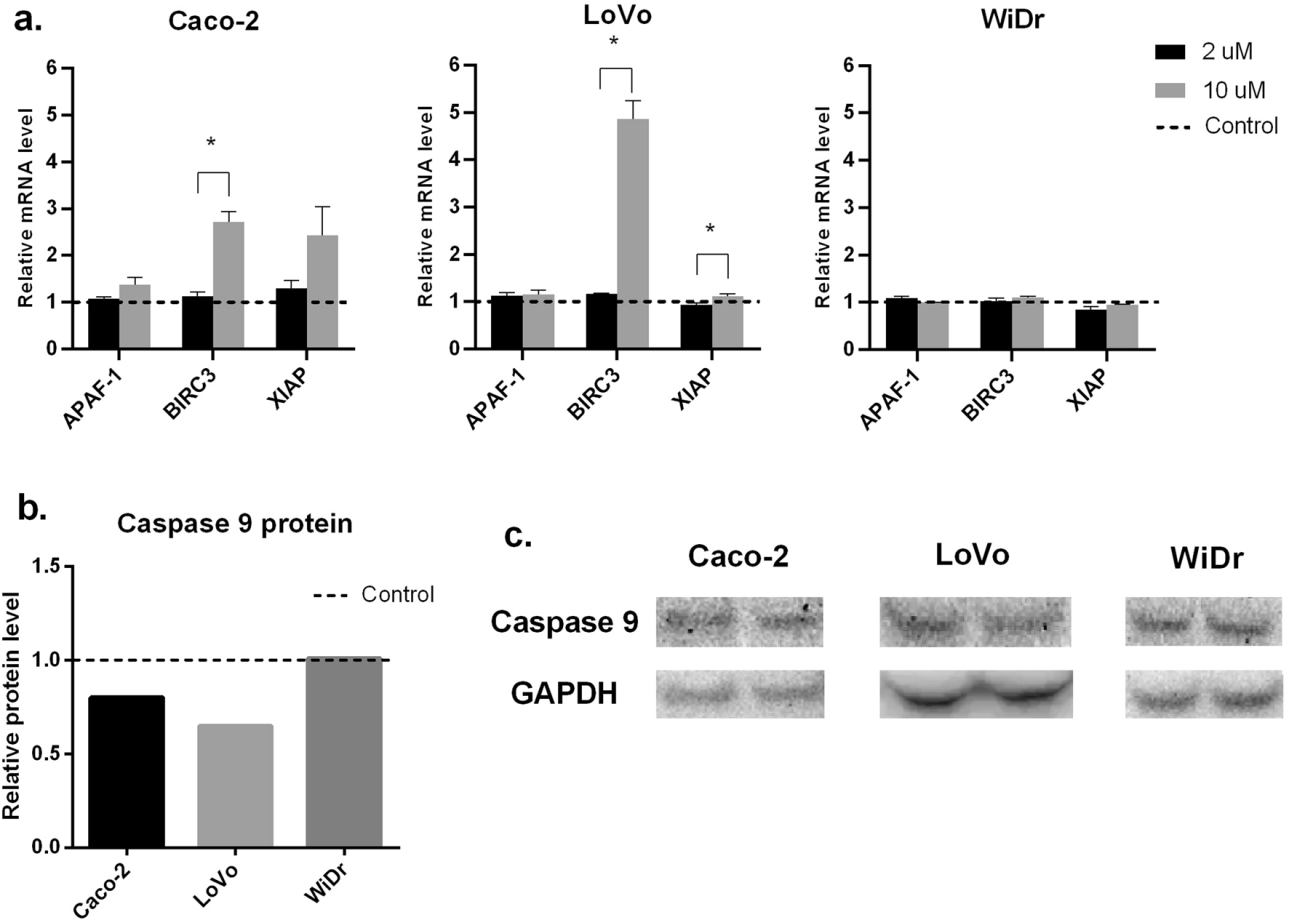
RT-PCR analysis of APAF-1, BIRC3, XIAP gene and Caspase 9 protein expression after HAMLET treatment of different cell lines. **A** RT-PCR analysis. **B** Western blot analysis of caspase 9 protein expression. **C** Western blot membrane band photo. *p < 0.05 when comparing between 2 μM and 10 μM. Control group data = 1—dotted line

To clarify the flow cytometry results, RT-PCR and WB analysis of apoptosis-related markers were performed. HAMLET complex did not affect BIRC2 or BIRC5 expression levels (data not demonstrated). There was also no noticeable increase in investigated gene expressions after treating cells with 2 μM HAMLET complex. When treating cells with 10 μM HAMLET complex Caco-2 cell line and LoVo cell line had a slight, although statistically insignificant, increase of pro-apoptotic APAF-1 gene expression (Caco-2—1,37 times; LoVo—1,16 times) and a high, statistically significant increase in anti-apoptotic BIRC3 (Caco-2—2,71 times; LoVo 4,86 times) and XIAP (Caco-2—2,43 times (statistically insignificant); LoVo—1,11 times (Statistically significant)) genes suggesting that apoptosis was being suppressed after HAMLET treatment (Fig. 5A). None of the apoptosis-related genes had any expression change in the WiDr cell line after treatment with HAMLET. To elucidate the functional response of cells to 10 μM HAMLET treatment, WB analysis of caspase 9 (cas9) protein was performed (Fig. 5B, C). Both Caco-2 and LoVo cell lines had decreased cas9 levels (Caco-2 0.8 times, LoVo—0.65 times). However, there was no change of cas9 in WiDr cell line. The results of WB conform with RT-PCR data demonstrating that increased levels of XIAP inhibited cas9 protein synthesis and in turn inhibited apoptosis of Caco-2 and LoVo cells.

### The effect of HAMLET on mitochondrial functions in colon cancer cells

**Fig. 6:**
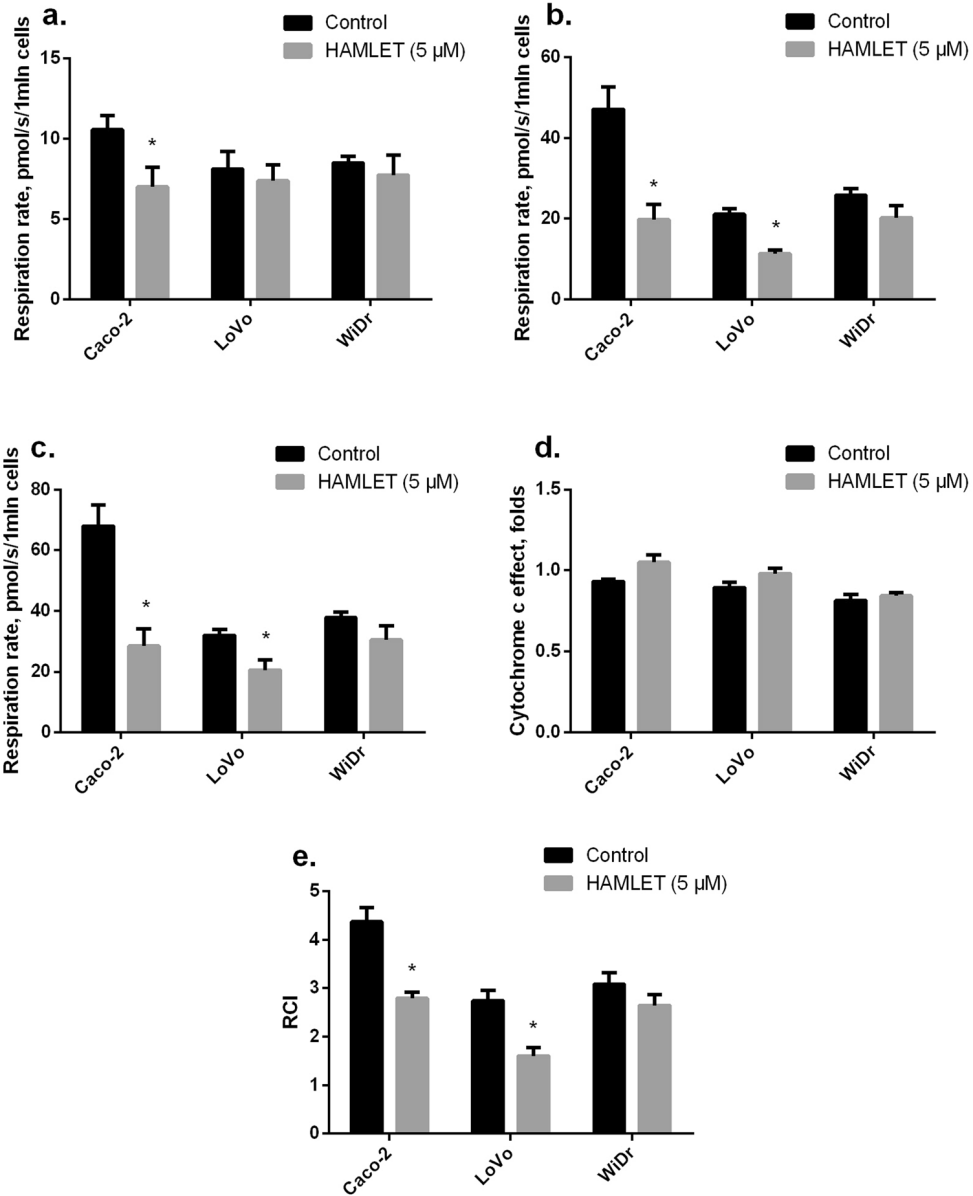
Effect of HAMLET on mitochondrial respiration and respiratory control index (RCI). Mitochondrial respiration rate was measured as described in “Methods”. **a** Mitochondrial non-phosphorylating (V_0_) respiration rate in the presence of 1 mln/mL of cells and glutamate (5 mM) plus malate (2 mM); **b** state 3 respiration rate in the presence of ADP (1 mM, VADP); **c** mitochondrial maximal respiration rate in the presence of succinate (12 mM, Vsucc); **d** mitochondrial respiration rate in the presence of cytochrome c (32 μM, Vcyt c); **e** mitochondrial respiratory control index (RCI) (VADP/V0)). *p < 0.05 as compared to the control group

We assessed the effect of HAMLET (5 µM) on mitochondrial functions in cancer cells (37 °C) by measuring mitochondrial respiration rate with glutamate/malate and succinate as substrates in three different cell lines (CaCo-2, LoVo and WiDr). HAMLET caused a statistically significant decrease in non-phosphorylating (V_0_) respiration rate (substrate glutamate/malate) by 38% in CaCo-2 cell lines but had no effect on non-phosphorylating (V_0_) respiration rate in LoVo and WiDr cell lines (Fig. 6A). Furthermore, mitochondrial State 3 (V_ADP_) respiration rate was reduced by 62%, 46% in CaCo and LoVo cell lines, respectively (*p*<0.05). HAMLET tended to decrease State 3 (V_ADP_) mitochondrial respiration rate in WiDr cell lines by 47% (*p*=0.057) (Fig. 6B). Moreover, pretreatment with HAMLET caused the decrease in maximal mitochondrial respiration (V_succ_) with complex II dependent substrate succinate by 62 % and 36 %, respectively, *p*<0.05, in CaCo-2 and LoVo cell lines, (Fig. 6C), and by 47% (*p*=0.054) in WiDr cell lines as compared to untreated cells (*p*<0.05). The addition of cytochrome c to mitochondria (Fig. 6D) showed that pretreatment of cancer cells with HAMLET has no effect on mitochondrial respiration rate V_cyt_. Therefore, these results suggest that HAMLET did not affect the mitochondrial outer membrane permeability. The respiratory control index (RCI, Fig. 6E) after pretreatment cells with HAMLET decreased by 33 and 19%, respectively, in Caco-2 and LoVo cell lines, as compared with untreated cells (*p*<0.05). However, there was no effect on RCI in WiDr cells. Pretreatment of cancer cells with HAMLET did not induce changes in carboxyatractyloside-dependent (V_CAT_) respiration rate (data not shown). Thus, HAMLET does not affect mitochondrial inner membrane permeability.

## Discussion

Treatment of advanced colorectal carcinoma is a clinical challenge for precision oncology due to the variation of molecular profiles, tumor microenvironment, and response to cytotoxic drugs and targeted agents.^6^ Despite positive outcomes published from selected trial patients, the effect on survival exceeding the specified study treatment remains uncertain (Modest et al. 2019). The concern is that the present first-line combination of chemotherapy and targeted treatment has little benefit and poor prognosis when applied to BRAF- and KRAS-mutation-bearing patients with mCRC (Li et al. 2020).

The relationship between mutations and susceptibility to treatment helps elucidate personalization trends. Before the study, we reviewed advanced CRC systemic treatment survival compared to the chemotherapy and biological therapy group. First, our hospital results revealed that the median survival of KRAS wild-type patients was statistically significantly longer by 2.5 months than KRAS mutation patients (33.0 months vs. 30.5 months) (Ilekis et al. 2017). These findings coincide with other published studies where median survival varies from 21 to 33 months (Stintzing et al. 2017; Modest et al. 2016). Second, compared to cohort groups, survival was not significantly different between the patients receiving and not receiving monoclonal antibodies. According to the trials, cetuximab significantly improves median overall survival by 3.5 months (Cutsem et al. 2011) and bevacizumab by 2 months (Hurwitz et al. 2013). Regrettably, we did not find any randomized trials with panitumumab. Nonetheless, this compound has a better effect than bevacizumab and is similar to cetuximab (McGregor et al. 2018). Furthermore, researchers from the University of Texas (Loree et al. 2018) suggested monoclonal antibodies as an adjunct to chemotherapy only for mCRC KRAS/BRAF double wild-type left-sided primary malignancies.

The advantage of biologic agents is relatively less profitable than expected, particularly when we consider possible side effects and select a suitable patient for therapy (Mármol et al. 2017). Currently, scientists concentrate on developing new personalized treatment options that are less aggressive and more effective than conventional ones. A novel anticancer drug, HAMLET, offers significant therapeutic potential with low toxicity (Ho et al. 2021). As demonstrated in this study, this complex efficiently suppressed three human colon cancer cells: double wild type; BRAF mutant; and KRAS mutant. Although this study did not confirm the initial hypothesis that KRAS and BRAF genes were associated with HAMLET, we found BRAF-mutant cell line resistance.

Compared to other all-natural mixtures found in food that can act as antitumor drugs, Genovese et al. (2020) reported gercumin. An active blend of curcumins inhibited two human colon cancer cells. At the same time, Fernández et al. (2021) tested five plant flavonoids for their potential as antitumor drugs against the same human CRC cell lines plus T84 (epithelial morphology, adenocarcinoma, metastasis in lung, KRAS mutant, BRAF wild type). Xanthohumol displayed the most significant antiproliferative activity of all flavonoids, even higher than the clinically used chemotherapy drug 5-fluorouracil.

Our flow cytometric analysis reported that HAMLET induced predominantly necrotic cell death. However, this disagrees with the literature data. A group of scientists from Lund University published that HAMLET causes mostly apoptosis-like death in tumor cells (Svanborg C et al. 2003). Presently, the suggested promising strategies for targeting CRC apoptotic pathways are direct activation of the extrinsic pathway by pro-apoptotic receptors, inactivation of BCL-2 proteins, caspase modification, and apoptosis protein inhibition (Abraha et al. 2016). HAMLET was shown to induce apoptosis via the mitochondrial pathway; according to the authors, apoptosis was initiated by releasing cytochrome C, activating caspase-2, -3, -9 and phosphatidylserine exposure (Ho et al. 2017). However, other studies indicated that caspase inhibitors, BCL-2 protein or p53 mutation did not prevent apoptosis, and apoptotic caspase cascade was not the leading cause of cell death (Hallgren et al. 2006; Mossberg et al. 2010; Gustafsson et al. 2009). The research question remains of cell death mechanism.

**Fig. 7:**
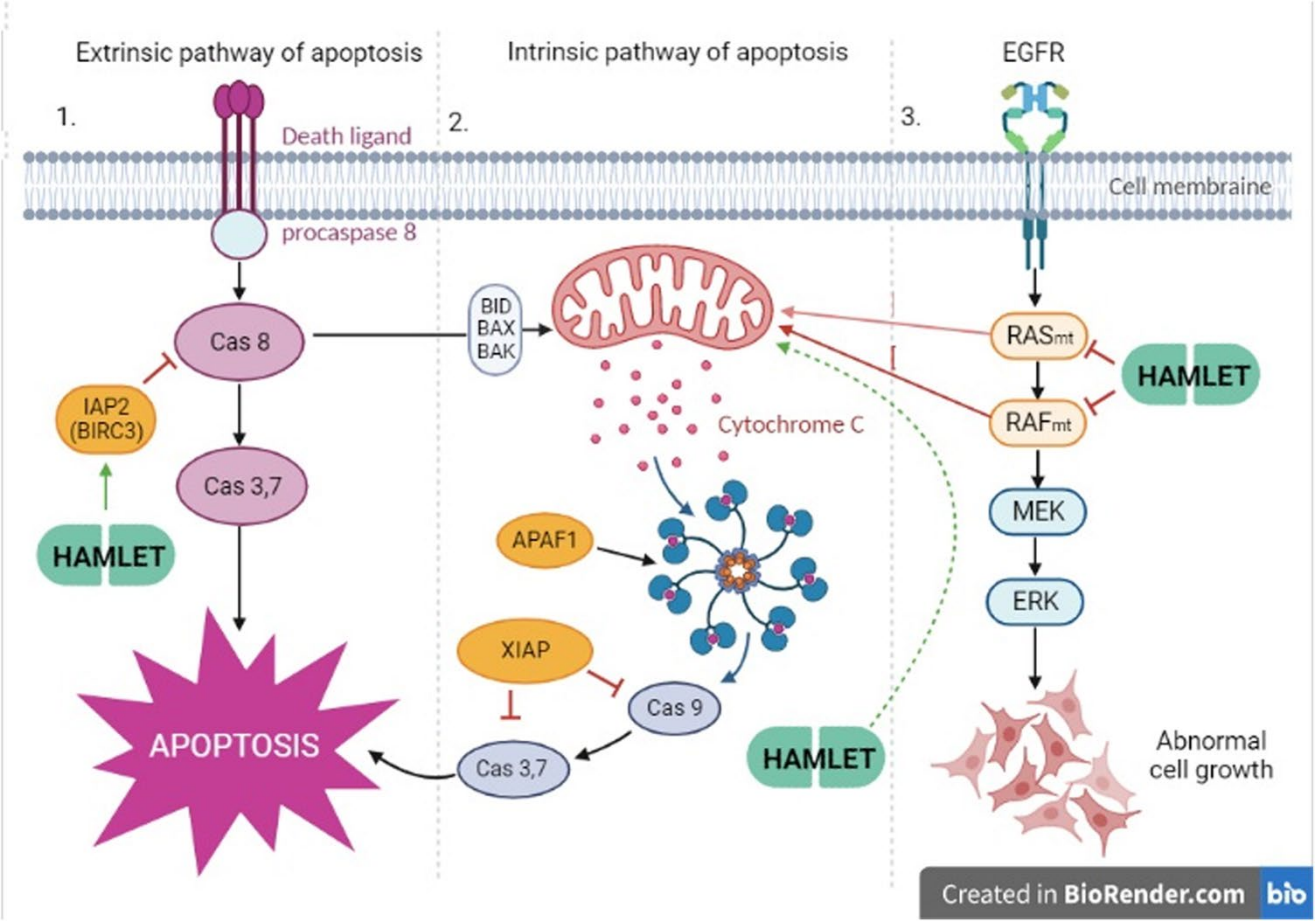
Mechanism of this study illustrating suppression of apoptosis and HAMLET targets. EGFR and its related proteins are involved in cell signaling pathways that control cell division and survival. EGFR-related RAF and RAS gene mutations cause proteins to produce higher than normal amounts in CRC. HAMLET inhibits oncogenic Ras and Braf activity which causes tumor death (Ho et al. 2017, 2016). KRAS/BRAF mutational status is also implicated in mitochondrial activity of CRC cancer (Rebane-Klemm et al. 2020). The relationship between HAMLET, RAS/RAF gene mutations and mitochondrial phenotype suggests that HAMLET affects cells through mitochondria depending on their activity. HAMLET is also known to activate apoptosis-like death mechanisms via intrinsic pathways (Boekema et al. 2015). However, APAF-1, an apoptotic protease activating factor that activates apoptosis, does not change after HAMLET treatment, and an increase of XIAP, one of the apoptosis-inhibiting proteins, shows that HAMLET does not cause canonical intrinsic pathway apoptosis. Up-regulation of BIRC3 (IAP2) also suggests that HAMLET treatment inhibits apoptosis in the extrinsic pathway as well, again showing that HAMLET causes cell death in a complex, non-apoptotic way which cannot be directly associated with KRAS or BRAF mutation

We investigated some apoptosis-related markers, such as the apoptosis-initiating gene APAF-1 and the apoptosis-inhibiting genes BIRC3 (IAP2), and XIAP, presented in the mechanistic scheme (Fig. 7). Our reverse transcription polymerase chain reaction gene expression analysis data revealed that HAMLET was not associated with the BIRC2 and BIRC5. The presence of non-mutant BRAF cells after HAMLET treatment allows the activation of more anti-apoptotic mechanisms through pathways such as increased BIRC3 and XIAP gene expression, which inhibits apoptosis through extrinsic or intrinsic activation. While pro-apoptotic gene APAF-1 expression has been slightly activated only in wild-type cells. BRAF-mutant WiDr cells do not have overexpression of any of these genes. In addition, we noticed that after HAMLET treatment, the BRAF-mutant WiDr cell line had a smaller number of necrotic cells than other investigated cells.

RT-PCR and flow cytometry findings indicate that there is no apoptotic death pathway, especially regarding WiDr cells. It is possible that the BRAF mutant could have necrotic or another death mechanism after HAMLET exposure. We hypothesized that different KRAS/BRAF mutational statuses of colorectal cancer cell lines affect the effectiveness of HAMLET anticancer and mitochondrial activity. Our clonogenicity analysis illustrates that the BRAF-mutant WiDr cell line was resistant to treatment with HAMLET.

Rebane-Klemm et al. (2020) revealed that mitochondrial activity varied between tumors of a similar genetic profile, and this is characteristic of KRAS and BRAF mutated and wild-type tumors. KRAS/BRAF mutational status is also implicated in mitochondrial activity of CRC with KRAS mutants having lower ADP-activated respiration rate than KRAS/BRAF wt and unchanged outer membrane permeability, suggesting an oxidative phenotype. BRAF mutant has an even lower respiration rate and altered outer membrane permeability suggesting glycolytic phenotype. Consequently, different metabolic resources can foresee a response to remedy, which helps with precision therapy.

Therefore, in the future, it would be appropriate to evaluate mitochondrial activity in a couple more BRAF normal/mutant cells after exposure to HAMLET. It could answer more mechanistic questions about the potential resistant tendency of BRAF mutation. In addition, it would be appropriate to analyze other markers related to necrosis or ferroptosis, which may occur due to lipid peroxidation.

A limitation of the study was that we investigated only three CRC cell lines. However, there was no clear link between the HAMLET cytotoxicity level and the bioenergetic profile provided. A more comprehensive analysis is required to detail further the effect of HAMLET on mitochondria and the glycolysis process. First, more CRC cell lines must be screened for the sensitivity of mitochondrial and glycolytic function to HAMLET treatment. Next, if the effects of HAMLET on mitochondria are observed, then the mechanism of action should be uncovered. A study on isolated rat liver mitochondria showed that HAMLET induced mitochondrial permeability transition, potentially leading to mitochondrial dysfunction and apoptosis (Köhler et al. 2001). This suggests possible testing if HAMLET affects permeability transition and related events, such as cytochrome c release. Another suggestion is that HAMLET might target mitochondrial ATP synthase (Boekema et al. 2015); thus, the sensitivity of this enzyme and other key enzyme complexes should also be assessed after HAMLET treatment. In addition to mitochondrial efficiency, cell glycolytic pathway sensitivity to HAMLET is crucial and might define the death/survival decision. HAMLET inhibits the glycolytic enzymes fructose bisphosphate aldolase and glyceraldehyde-3-phosphate dehydrogenase in bacteria (Roche-Hakansson et al. 2019); thus, similar glycolysis suppressing activity could also take place in eukaryotic cells. The precise definition of HAMLET-sensitive and not-sensitive members of mitochondrial and glycolytic energetic pathways will allow the creation of a strategy for patient stratification and identification of additional treatment targets.

To the best of our knowledge, this is the first study evaluating how KRAS/BRAF mutation status affects HAMLET anticancer activity. One of the recently described HAMLET efficiency regulating mechanisms is related to alpha-helical- or beta-sheet domains of alpha-lactalbumin in heat shock proteins, resulting in an immediate death response or a delay due to transient accumulation of the HAMLET complex in lysosomes (Nadeem A et al. 2019). However, this finding provides no direct clues to the relationship between KRAS/BRAF pathway and energetic metabolism. Nevertheless, the complex is actively exploited because of its prominent selective toxicity to cancer cells. A group of scientists from Lund University has recently published the first HAMLET data on a single-center, placebo-controlled, double-blinded randomized phase I/II interventional clinical trial of non-muscle invasive bladder cancer. Researchers concluded that intravesical inoculation of alpha1-oleate was safe and effective in patients with bladder cancer (Brisuda et al. 2021). After this successful trial, the Lund University group shared other trial ideas of having this proteolipid compound in drinking water as prevention. Targeting early, locally growing tumors is essential to reduce tumor progression and metastatic disease risk (Smith 2013). A further research direction would be testing the efficiency of HAMLET on fresh surgically resected human colorectal tumor biopsies ex vivo (Novo et al. 2017) to identify patient responses together with analysis of tumor bioenergetic profiles for a patient stratification strategy.

## Conclusions

HAMLET exhibits irreversible cytotoxicity on human CRC cells in a dose-dependent manner, leading to necrotic cell death and inhibiting the extrinsic apoptosis pathway. BRAF-mutant cell line is more resistant than other type lines. HAMLET decreased mitochondrial respiration and ATP synthesis in CaCo-2 and LoVo cell lines but did not affect WiDr cells’ respiration. Pretreatment of cancer cells with HAMLET has no impact on mitochondrial outer and inner membrane permeability.

## Data Availability

The data sets analyzed during the current study are available from the corresponding author on reasonable request.

## References

[CR1] Aasebø K, Dragomir A, Sundström M et al (2020) CDX2: a prognostic marker in metastatic colorectal cancer defining a better *BRAF* mutated and a worse *KRAS* mutated subgroup. Front Oncol 10:8. 10.3389/fonc.2020.00008 32117703 10.3389/fonc.2020.00008 PMC7026487

[CR2] Abraha AM, Ketema EB (2016) Apoptotic pathways as a therapeutic target for colorectal cancer treatment. World J Gastrointest Oncol 8(8):583–591. 10.4251/wjgo.v8.i8.583 27574550 10.4251/wjgo.v8.i8.583 PMC4980648

[CR3] Ahmed D, Eide PW, Eilertsen IA et al (2013) Epigenetic and genetic features of 24 colon cancer cell lines. Oncogenesis 2(9):e71. 10.1038/oncsis.2013.35 24042735 10.1038/oncsis.2013.35 PMC3816225

[CR4] André T, Shiu KK, Kim TW et al (2020) Pembrolizumab in microsatellite-instability-high advanced colorectal cancer. N Engl J Med 383(23):2207–2218. 10.1056/nejmoa2017699 33264544 10.1056/NEJMoa2017699

[CR5] Arcila M, Lau C, Nafa K, Ladanyi M (2011) Detection of KRAS and BRAF mutations in colorectal carcinoma roles for high-sensitivity locked nucleic acid-PCR sequencing and broad-spectrum mass spectrometry genotyping. J Mol Diagn 13(1):64–73. 10.1016/j.jmoldx.2010.11.005 21227396 10.1016/j.jmoldx.2010.11.005 PMC3070595

[CR6] Boekema EJ (2015) A passive function of mitochondrial ATP synthase: target for tumor killer HAMLET. J Mol Biol 427(10):1863–1865. 10.1016/j.jmb.2015.03.003 25754830 10.1016/j.jmb.2015.03.003

[CR7] Brisuda A, Ho JCS, Kandiyal PS et al (2021) Bladder cancer therapy using a conformationally fluid tumoricidal peptide complex. Nat Commun 12(1):3427. 10.1038/s41467-021-23748-y 34103518 10.1038/s41467-021-23748-y PMC8187399

[CR8] Cohen R, Liu H, Fiskum J et al (2021) BRAF V600E mutation in first-line metastatic colorectal cancer: an analysis of individual patient data from the ARCAD database. J Natl Cancer Inst 113(10):1386–1395. 10.1093/jnci/djab042 33734401 10.1093/jnci/djab042 PMC7617278

[CR9] Cooper S, Bouvy JC, Baker L et al (2020) How should we assess the clinical and cost effectiveness of histology independent cancer drugs? BMJ 368:l6435. 10.1136/bmj.l6435 31896539 10.1136/bmj.l6435

[CR10] Douillard JY, Oliner KS, Siena S et al (2013) Panitumumab-FOLFOX4 treatment and RAS mutations in colorectal cancer. N Engl J Med 369(11):1023–1034. 10.1056/nejmoa1305275 24024839 10.1056/NEJMoa1305275

[CR11] Fernández J, Silván B, Entrialgo-Cadierno R et al (2021) Antiproliferative and palliative activity of flavonoids in colorectal cancer. Biomed Pharmacother 143:112241. 10.1016/j.biopha.2021.112241 34649363 10.1016/j.biopha.2021.112241

[CR12] Fujii S, Magliocco AM, Kim J et al (2020) International harmonization of provisional diagnostic criteria for *ERBB2*-amplified metastatic colorectal cancer allowing for screening by next-generation sequencing panel. JCO Precis Oncol 4:6–19. 10.1200/po.19.00154 35050726 10.1200/PO.19.00154

[CR13] Genovese S, Epifano F, Preziuso F et al (2020) Gercumin synergizes the action of 5-fluorouracil and oxaliplatin against chemoresistant human cancer colon cells. Biochem Biophys Res Commun 522(1):95–99. 10.1016/j.bbrc.2019.11.068 31740005 10.1016/j.bbrc.2019.11.068

[CR14] Gustafsson L, Aits S, Onnerfjord P, Trulsson M, Storm P, Svanborg C (2009) Changes in proteasome structure and function caused by HAMLET in tumor cells. PLoS ONE 4(4):e5229. 10.1371/journal.pone.0005229 19365565 10.1371/journal.pone.0005229 PMC2664966

[CR15] Hallgren O, Gustafsson L, Irjala H, Selivanova G, Orrenius S, Svanborg C (2006) HAMLET triggers apoptosis but tumor cell death is independent of caspases, Bcl-2 and p53. Apoptosis 11(2):221–233. 10.1007/s10495-006-3607-7 16502260 10.1007/s10495-006-3607-7

[CR16] Hamers P, Bos ACRK, May AM, Punt CJA, Koopman M, Vink GR (2019) Recent changes in overall survival of real-life stage IV colorectal cancer patients. J Clin Oncol 37(15):3522–3522. 10.1200/jco.2019.37.15_suppl.3522

[CR17] Ho JC, Nadeem A, Rydström A, Puthia M, Svanborg C (2016) Targeting of nucleotide-binding proteins by HAMLET–a conserved tumor cell death mechanism. Oncogene 35(7):897–907. 10.1038/onc.2015.144 26028028 10.1038/onc.2015.144

[CR18] Ho JCS, Ambite I, Mok KH, Babjuk M, Svanborg C (2021) A scientific journey from discovery to validation of efficacy in cancer patients: HAMLET and alpha1-oleate. Mol Cell Oncol 8(5):1974278. 10.1080/23723556.2021.1974278 34859140 10.1080/23723556.2021.1974278 PMC8632289

[CR19] Ho JCS, Nadeem A, Svanborg C (2017) HAMLET—A protein-lipid complex with broad tumoricidal activity. Biochem Biophys Res Commun 482(3):454–458. 10.1016/j.bbrc.2016.10.092 28212731 10.1016/j.bbrc.2016.10.092

[CR20] Hurwitz HI, Tebbutt NC, Kabbinavar F et al (2013) Efficacy and safety of bevacizumab in metastatic colorectal cancer: pooled analysis from seven randomized controlled trials. Oncologist 18(9):1004–1012. 10.1634/theoncologist.2013-0107 23881988 10.1634/theoncologist.2013-0107 PMC3780632

[CR21] Ilekis M, Korobeinikova E (2017) RAS onkogenų šeimos mutacijų įtaka gydymo biologine terapija pasirinkimui ir pacientų išgyvenamumui, sergant išplitusiu gaubtinės ar tiesiosios žarnos vėžiu. Jaunųjų mokslininkų ir tyrėjų konferencija [69-oji]–JMTK: tezių knyga 2017: [2017 m. gegužės 17–19 d., Kaunas]/Lietuvos sveikatos mokslų universiteto (LSMU) Studentų mokslinė draugija (SMD) https://www.lsmuni.lt/cris/handle/20.500.12512/96111 (accessed 2023–01–08).

[CR22] Kamijima T, Ohmura A, Sato T et al (2008) Heat-treatment method for producing fatty acid-bound alpha-lactalbumin that induces tumor cell death. Biochem Biophys Res Commun 376(1):211–214. 10.1016/j.bbrc.2008.08.127 18774773 10.1016/j.bbrc.2008.08.127

[CR23] Kim JC, Bodmer WF (2022) Genomic landscape of colorectal carcinogenesis. J Cancer Res Clin Oncol 148(3):533–545. 10.1007/s00432-021-03888-w 35048197 10.1007/s00432-021-03888-w PMC11800883

[CR24] Köhler C, Gogvadze V, Håkansson A, Svanborg C, Orrenius S, Zhivotovsky B (2001) A folding variant of human alpha-lactalbumin induces mitochondrial permeability transition in isolated mitochondria. Eur J Biochem 268(1):186–191. 10.1046/j.1432-1327.2001.01870.x 11121120 10.1046/j.1432-1327.2001.01870.x

[CR25] Li ZN, Zhao L, Yu LF, Wei MJ (2020) *BRAF* and *KRAS* mutations in metastatic colorectal cancer: future perspectives for personalized therapy. Gastroenterol Rep (oxf) 8(3):192–205. 10.1093/gastro/goaa022 32665851 10.1093/gastro/goaa022 PMC7333923

[CR26] Loree JM, Pereira AAL, Lam M et al (2018) Classifying colorectal cancer by tumor location rather than sidedness highlights a continuum in mutation profiles and consensus molecular subtypes. Clin Cancer Res 24(5):1062–1072. 10.1158/1078-0432.ccr-17-2484 29180604 10.1158/1078-0432.CCR-17-2484 PMC5844818

[CR27] Mármol I, Sánchez-de-Diego C, Pradilla Dieste A, Cerrada E, Rodriguez Yoldi MJ (2017) Colorectal carcinoma: a general overview and future perspectives in colorectal cancer. Int J Mol Sci 18(1):197. 10.3390/ijms18010197 28106826 10.3390/ijms18010197 PMC5297828

[CR28] McGregor M, Price TJ (2018) Panitumumab in the treatment of metastatic colorectal cancer, including wild-type RAS. KRAS NRAS mCRC Fut Oncol 14(24):2437–2459. 10.2217/fon-2017-0711 10.2217/fon-2017-0711 29737864

[CR29] Modest DP, Pant S, Sartore-Bianchi A (2019) Treatment sequencing in metastatic colorectal cancer. Eur J Cancer 109:70–83. 10.1016/j.ejca.2018.12.019 30690295 10.1016/j.ejca.2018.12.019

[CR30] Modest DP, Ricard I, Heinemann V et al (2016) Outcome according to KRAS-, NRAS- and BRAF-mutation as well as KRAS mutation variants: pooled analysis of five randomized trials in metastatic colorectal cancer by the AIO colorectal cancer study group. Ann Oncol 27(9):1746–1753. 10.1093/annonc/mdw261 27358379 10.1093/annonc/mdw261 PMC4999563

[CR31] Molinari C, Marisi G, Passardi A, Matteucci L, De Maio G, Ulivi P (2018) Heterogeneity in colorectal cancer: a challenge for personalized medicine? Int J Mol Sci 19(12):3733. 10.3390/ijms19123733 30477151 10.3390/ijms19123733 PMC6321493

[CR32] Mossberg AK, Puchades M, Halskau Ø et al (2010) HAMLET interacts with lipid membranes and perturbs their structure and integrity. PLoS ONE 5(2):e9384. 10.1371/journal.pone.0009384 20186341 10.1371/journal.pone.0009384 PMC2826418

[CR33] Nadeem A, Ho JCS, Tran TH et al (2019) Beta-sheet-specific interactions with heat shock proteins define a mechanism of delayed tumor cell death in response to HAMLET. J Mol Biol 431(14):2612–2627. 10.1016/j.jmb.2019.05.00710.1016/j.jmb.2019.05.007 31082436 10.1016/j.jmb.2019.05.007

[CR34] Novo SM, Wedge SR, Stark LA (2017) Ex vivo treatment of patient biopsies as a novel method to assess colorectal tumour response to the MEK1/2 inhibitor, Selumetinib. Sci Rep 7(1):12020. 10.1038/s41598-017-12222-9 28931905 10.1038/s41598-017-12222-9 PMC5607258

[CR35] Oikonomou E, Koustas E, Goulielmaki M, Pintzas A (2014) BRAF vs RAS oncogenes: are mutations of the same pathway equal? Differential signalling and therapeutic implications. Oncotarget 5(23):11752–11777 25361007 10.18632/oncotarget.2555 PMC4322985

[CR36] Rebane-Klemm E, Truu L, Reinsalu L et al (2020) Mitochondrial Respiration in *KRAS* and *BRAF* mutated colorectal tumors and polyps. Cancers (basel) 12(4):815. 10.3390/cancers12040815 32231083 10.3390/cancers12040815 PMC7226330

[CR37] Roche-Hakansson H, Vansarla G, Marks LR, Hakansson AP (2019) The human milk protein-lipid complex HAMLET disrupts glycolysis and induces death in *Streptococcus pneumoniae*. J Biol Chem 294(51):19511–19522. 10.1074/jbc.RA119.009930 31694917 10.1074/jbc.RA119.009930 PMC6926454

[CR38] Smith K (2013) Therapy: HAMLET takes a leading role on the colorectal cancer stage. Nat Rev Gastroenterol Hepatol 10(3):126. 10.1038/tp.2013.27 23399531 10.1038/nrgastro.2013.27

[CR39] Stintzing S, Modest DP, Rossius L et al (2016) FOLFIRI plus cetuximab versus FOLFIRI plus bevacizumab for metastatic colorectal cancer (FIRE-3): a post-hoc analysis of tumour dynamics in the final RAS wild-type subgroup of this randomised open-label phase 3 trial. Lancet Oncol 17(10):1426–1434. 10.1016/s1470-2045(16)30269-8 27575024 10.1016/S1470-2045(16)30269-8

[CR40] Sung H, Ferlay J, Siegel RL et al (2021) Global cancer statistics 2020: GLOBOCAN estimates of incidence and mortality worldwide for 36 Cancers in 185 Countries. CA Cancer J Clin 71(3):209–249. 10.3322/caac.21660 33538338 10.3322/caac.21660

[CR41] Svanborg C, Agerstam H, Aronson A et al (2003) HAMLET kills tumor cells by an apoptosis-like mechanism–cellular, molecular, and therapeutic aspects. Adv Cancer Res 88:1–29. 10.1016/s0065-230x(03)88302-1 12665051 10.1016/s0065-230x(03)88302-1

[CR42] Tougeron D, Lecomte T, Pagès JC et al (2013) Effect of low-frequency KRAS mutations on the response to anti-EGFR therapy in metastatic colorectal cancer. Ann Oncol 24(5):1267–1273. 10.1093/annonc/mds620 23293113 10.1093/annonc/mds620

[CR43] Van Cutsem E, Köhne CH, Láng I et al (2011) Cetuximab plus irinotecan, fluorouracil, and leucovorin as first-line treatment for metastatic colorectal cancer: updated analysis of overall survival according to tumor KRAS and BRAF mutation status. J Clin Oncol 29(15):2011–2019. 10.1200/jco.2010.33.5091 21502544 10.1200/JCO.2010.33.5091

